# Genetic structuring, dispersal and taxonomy of the high-alpine populations of the *Geranium arabicum/kilimandscharicum* complex in tropical eastern Africa

**DOI:** 10.1371/journal.pone.0178208

**Published:** 2017-05-26

**Authors:** Tigist Wondimu, Abel Gizaw, Felly M. Tusiime, Catherine A. Masao, Ahmed A. Abdi, Yan Hou, Sileshi Nemomissa, Christian Brochmann

**Affiliations:** 1 Department of Plant Biology & Biodiversity Management, College of Natural Sciences, Addis Ababa University, Addis Ababa, Ethiopia; 2 Natural History Museum, University of Oslo, Blindern, Oslo, Norway; 3 Department of Forestry and Tourism, School of Forestry, Geographical and Environmental Sciences, Makerere University, Kampala, Uganda; 4 University of Dar es Salaam, Institute of Resource Assessment, Dar es Salaam, Tanzania; 5 National Museums of Kenya, Nairobi, Kenya; National Cheng Kung University, TAIWAN

## Abstract

The scattered eastern African high mountains harbor a renowned and highly endemic flora, but the taxonomy and phylogeographic history of many plant groups are still insufficiently known. The high-alpine populations of the *Geranium arabicum/kilimandscharicum* complex present intricate morphological variation and have recently been suggested to comprise two new endemic taxa. Here we aim to contribute to a clarification of the taxonomy of these populations by analyzing genetic (AFLP) variation in range-wide high-alpine samples, and we address whether hybridization has contributed to taxonomic problems. We identified only two genetic groups. One corresponded to *G*. *kilimandscharicum*, which has been reported as exclusively high-alpine and confined to the eastern Rift mountains in East Africa. The other corresponded to *G*. *arabicum*, reported from lower altitudes on the same mountains as well as from a wide altitudinal span in Ethiopia and on the western Rift mountains in East Africa. The four populations analyzed of a recently described species from the Bale Mts in Ethiopia were admixed, indicating that they result from recent long-distance dispersal of *G*. *kilimandscharicum* from East Africa followed by hybridization with local *G*. *arabicum* in naturally disturbed habitats. Some admixture between the two genetic groups was also inferred on other mountains, supporting earlier suggestions of introgression based on morphology. We did not find support for recognition of the recently suggested new subspecies of *G*. *arabicum* in Ethiopia. Interestingly, the high-alpine *G*. *kilimandscharicum* lacked clear geographic structuring, suggesting a recent history of colonization of the different mountains or extensive intermountain gene flow.

## Introduction

The flora of the scattered African high mountains is renowned in biogeography for its peculiar life forms, many endemics, and close relationships to temperate floras in other parts of the world. Although the tropical afro-alpine flora is quite poor in terms of species number, with only 521 species recognized in a recent enumeration [1], not only the bio- and phylogeographic history but also the taxonomy of many plant groups are still insufficiently known. There is a clear need for renewed efforts including more extensive field sampling to clarify the taxonomy of such groups, to test delimitation of species based on genetic data, and to address the relative importance of processes such as intermountain divergence, long-distance dispersal and hybridization in this fascinating system of fragmented ‘sky islands’ [2–4].

Recent phylogeographic studies have shown that afro-alpine plant species or species complexes can show more or less distinct genetic structuring corresponding to individual mountains or mountain groups, suggesting a long history of isolation after initial colonization(*Erica trimera*, [2]; *Deschampsia cespitosa*, [3]; *Carex monostachya*, [5]). Several studies have shown however that intermountain gene flow may be more common than previously thought(*Lobelia gibberoa*,[6]; *Trifolium cryptopodium*, [4]). In some species there is no or only little geographic structuring of the genetic diversity, suggesting recent colonization and/or extensive recent dispersal(*Erica arborea*,[2]; *Koeleria capensis*, [3]) and in others, there is clear evidence for intermountain dispersal followed by hybridization between divergent lineages (*Carex* spp., [5]; *Carduus schimperi*, [4]).Because the species and species complexes studied to date show a surprising variety of phylogeographic histories, more case studies are needed to assess to what degree there are general patterns in the history of the enigmatic afro-alpine flora, and to assess the influence of processes such as intermountain dispersal and hybridization on the morphology of afro-alpine plants.

A typical example showing intricate morphological variation and poorly resolved taxonomy is provided by the eastern African populations of the *Geranium arabicum/kilimandscharicum* complex. Typical *G*. *arabicum*has pentagonal leaves and flowers in pairs, whereas *G*. *kilimandscharicum* has reniform leaves and solitary flowers. However, morphological intermediates do occur and are thought to result from introgression between the two species [7], and it has been suggested that it might be most appropriate to recognize them at the subspecies level [8].These small perennial herbs grow in montane and alpine grasslands and on rocky ground with open vegetation, and show the typical *Geranium* mode of short-distance dispersal by ejecting their mericarps after explosive curling of the awns [8].The complex morphological variation in this group resulted in description of several species by early taxonomists, but only two were accepted in the treatments of [9] and [8]:They reported *G*. *kilimandscharicum* Engl. as exclusively high-alpine and confined to the eastern Rift mountains in East Africa (Mt Kilimanjaro (type), Mt Meru, Mt Kenya, Aberdare Mts, Mt Elgon; [9]; [7]. The other species, *G*. *arabicum* Forssk., is typified from Yemen and reported as widespread in tropical African highlands between 1000 and 4000 m [7]; [8]. The two species co-occur on the eastern Rift mountains in East Africa, where *G*. *arabicum*usually occurs at lower altitudes (recorded up to 3950 m, but usually well below this altitude) and *G*. *kilimandscharicum* usually at higher altitudes (3200–4400 m; [7]).

Recently, a third species of the complex was tentatively described as endemic to the Bale mountains in *Flora of Ethiopia and Eritrea*, listed as ‘*Geranium* sp.’ [8]. This species also has reniform leaves and solitary flowers, but it appears conspicuously different from *G*. *kilimandscharicum* by its acaulescent stem and by its exceptionally overlapping leaf lobes, which make the leaves look ‘double’. It is only known from a few high-alpine (4150–4200 m) localities where the ground has been disturbed by activities of the giant mole rat or by frost heaving. In addition, a tentative new endemic subspecies of *G*. *arabicum* was listed in the *Flora of Ethiopia and Eritrea*, referred to as subspecies ‘*Ash 1711*’ based on a collection made in the Bale Mts [8]. This subspecies was reported from afro-alpine grasslands also on several other mountains in Ethiopia and Eritrea, typically at higher altitudes (2900–4000 m) than subspecies *arabicum*(1300–3650 m). Interestingly, [8] pointed out that some of the material referred to this subspecies was morphologically similar to the East African *G*. *kilimandscharicum*. A third subspecies, *G*. *arabicum* ssp.*latistipulatum* (A.Rich.) Kokwaro, has been recognized from low altitudes (1000–3150 m) in *Erica* forests both in East Africa and Ethiopia. The main leaf lobes of ssp. *latistipulatum* are deeply pinnatisect into narrowly oblong segments separated by wide sinuses. The other two subspecies have less dissected leaf lobes and narrow sinuses, but ssp. ‘*Ash 1711*’ differs from ssp. *arabicum* by having more reniform (vs pentagonal) leaf outline and wedge-shaped (vs rhombic) outline of the main leaf lobes [8].

Further studies are clearly needed to clarify the variation in the eastern African populations of the *Geranium arabicum/kilimandscharicum* complex. Here we aim to contribute to a clarification of the taxonomy of the high-alpine populations of this complex by analyzing genetic (AFLP) variation in nearly range-wide samples, and also to address their phylogeographic history. In particular, we ask whether hybridization has contributed to taxonomic problems in this group. For logistic reasons we were not able to cover the lower-altitude subspecies in the widespread *G*. *arabicum*, because our sampling was carried out as part of a larger project restricted to high-alpine areas.

## Materials and methods

### Materials

**Fig 1:**
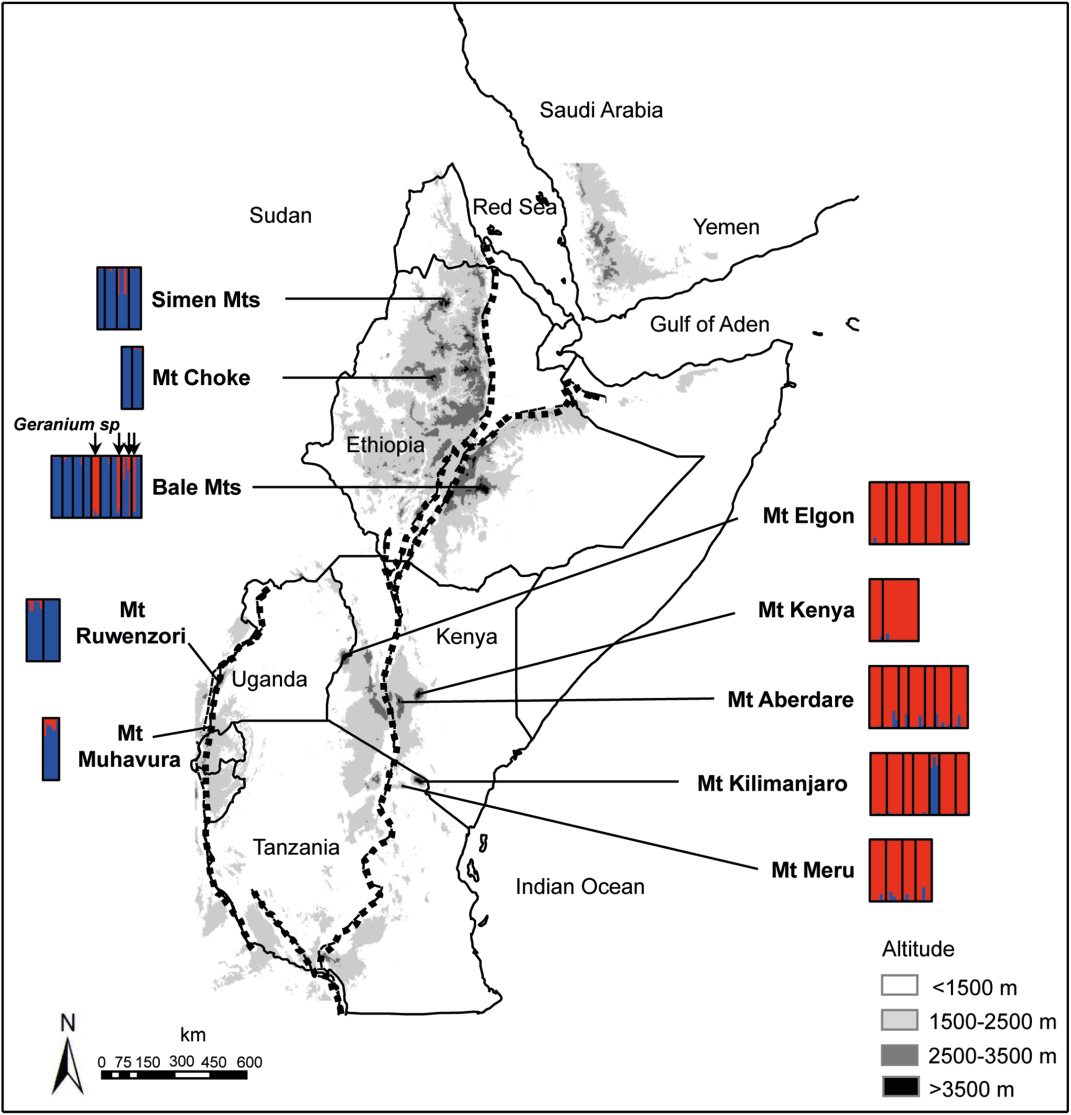
Sampling sitesand main genetic structuring in eastern African high-altitude populations of *Geranium* based on the total AFLP dataset of 211 individual plants (45 populations) successfully analysed. Colours represent the two main genetic groups inferred in the STRUCTURE analyses (blue: *G*. *arabicum* group, red: *G*. *kilimandscharicum* group).

We carried out field work in 10 mountain systems in Ethiopia, Kenya, Tanzania and Uganda (Fig 1, Table 1).Permits were obtained from the responsible authorities in each country: The Ethiopian Wildlife Conservation Authority (EWCA) for the Simen and Bale Mountains National Parks; the Kenyan Wildlife Service (KWS) for Mount Kenya; the Tanzanian Wildlife Authority (TAWA) for Mt. Kilimanjaro and Mt. Arusha; and the Ugandan Wildlife Authority (UWA) for Mt. Ruwenzori and Mt. Muhavura. Leaf samples were collected from five individual plants within an area of100 m × 100 m, taken to represent a single population, and dried in silica gel. We aimed to cover the morphological variation observed in the alpine zone of each mountain and collected a total of 289 plants from 51populations. On the basis of their morphology, 28 populations were tentatively referred to *G*. *kilimandscharicum*, 10 to *G*. *arabicum*ssp.*arabicum*, six to *G*. *arabicum* ssp. ‘*Ash 1711*’, and seven to *Geranium* sp.sensu [8]. We were not able to collect *G*. *arabicum* ssp. *latistipulatum*, which is recorded only from a few subalpine areas in East Africa(below 2800 m, [7]) and Ethiopia (below 3150 m; [8]). Our sampling represented all mountains recorded for the eastern East African endemic *G*. *kilimandscharicum* [9]; [7], the single mountain range (Bale Mts) recorded for the tentative Ethiopian endemic *Geranium* sp. [8], and two of the mountain ranges (Bale and Choke Mts) recorded for the tentative Ethiopian endemic *G*. *arabicum* ssp. ‘*Ash 1711*’ (Fig 1). For *G*. *arabicum* ssp. *arabicum*, our sampling included its high-alpine occurrences along the western branch of the Rift Valley in East Africa (Mt Ruwenzori and Mt Muhavura) and in Ethiopia (Bale and Simen), outside the recorded range of *G*. *kilimandscharicum*. In addition, we sampled one population referred to *G*. *arabicum* ssp. *arabicum* from the montane forest zone in Mt Kilimanjaro (< 3000 m; Fig 1). Three of the five plants from each population were pressed and deposited in the following herbaria: one in the Natural History Museum, University of Oslo (O), Norway; one in the National Herbarium, Addis Ababa University (ETH), Ethiopia; and the third voucher was deposited according to country of collection, i.e., in the East African Herbarium (EA), Kenya, at the Sokoine University of Agriculture (SUA), Tanzania, or Makerere University Herbarium (MHU), Uganda.

**Table 1:** Sampling data for the eastern African high-altitude populations of *Geranium* successfully analyzed for AFLPs. *P*(*%*): Percentage of polymorphic loci; *D*: Nei’s gene diversity following [10]; DW: frequency-down-weighted marker value as a measure of genetic rarity following [11].The three populations tentatively identified as *Geranium* sp. sensu [8] with numbers in bold consisted of plants belonging to both genetic groups. Database number refers to the DNA Bank at the Natural History Museum, University of Oslo.

Genetic group/tentative taxon identification	Database number	Population number	Country	Mountain: locality	Latitude	Longitude	Altitude (m)	*n*	*P*(*%*)	*D*	DW
***G*. *kilimandscharicum* genetic group**											
*Geranium* sp.	O-DP-32859–32860	**ET0954**	Ethiopia	Bale Mts: Batu	6.8500	39.8532	4116	2	4.24	0.042	2.83
*Geranium* sp.	O-DP-32951	**ET0974**	Ethiopia	Bale Mts: Angaso	6.9049	39.9046	3875	1	-	-	-
*Geranium* sp.	O-DP-33140	**ET1030**	Ethiopia	Bale Mts: Angaso	6.8931	39.8974	3875	1	-	-	-
*Geranium* sp.	O-DP-31734–31738	ET0677	Ethiopia	Bale Mts: Sanetti		_	4050	5	8.94	0.041	2.56
*G*. *kilimandscharicum*	O-DP-34837–34841	KN0028	Kenya	Mt Elgon: S of Mt Koitobos	1.1057	34.6018	3915	5	4.71	0.021	0.99
*G*. *kilimandscharicum*	O-DP-35100–35102	KN0097	Kenya	Mt Elgon: Mt Koitobos	1.1240	34.5903	3953	3	2.35	0.016	0.81
*G*. *kilimandscharicum*	O-DP-35466, 35468–35470	KN0180	Kenya	Mt Elgon: Caldera	1.1180	34.5867	4043	4	4.24	0.022	1.01
*G*. *kilimandscharicum*	O-DP-35654–35658	KN0225	Kenya	Mt Elgon: Near camp site at end of car road	1.0900	34.6181	3670	5	8.00	0.034	1.58
*G*. *kilimandscharicum*	O-DP-35790–35794	KN0256	Kenya	Mt Elgon: E of Mt Koitobos	1.1083	34.6061	3864	5	7.06	0.032	1.25
*G*. *kilimandscharicum*	O-DP-35904, 35906–35908	KN0279	Kenya	Mt Elgon: NE of Mt Koitobos	1.1029	34.6131	3979	4	3.76	0.021	0.88
*G*. *kilimandscharicum*	O-DP-36093, 36095–36097	KN0320	Kenya	Mt Elgon: S of Mt Koitobos	1.1007	34.6215	3629	4	6.59	0.034	1.72
*G*. *kilimandscharicum*	O-DP-27381, 27383–27385	KN0464	Kenya	Aberdare Mts: Mt Kinangop area	-0.5590	36.7168	3033	4	10.59	0.056	3.16
*G*. *kilimandscharicum*	O-DP-27456–27460	KN0480	Kenya	Aberdare Mts: Mt Kinangop area	-0.5459	36.7182	3074	5	11.29	0.048	1.79
*G*. *kilimandscharicum*	O-DP-27475–27477	KN0484	Kenya	Aberdare Mts: Mt Kinangop area	-0.5530	36.7204	3068	3	7.06	0.047	1.73
*G*. *kilimandscharicum*	O-DP-27727–27731	KN0542	Kenya	Aberdare Mts: Mt Satima, peak	-0.3037	36.6177	3997	5	5.18	0.022	1.39
*G*. *kilimandscharicum*	O-DP-28146–28148	KN0643	Kenya	Aberdare Mts: Mt Satima area	-0.3333	36.6415	3584	3	4.94	0.033	1.12
*G*. *kilimandscharicum*	O-DP-28165–28169	KN0647	Kenya	Aberdare Mts: Mt Satima area	-0.3515	36.6500	3618	5	10.82	0.047	2.72
*G*. *kilimandscharicum*	O-DP-28185–28189	KN0651	Kenya	Aberdare Mts: Mt Satima area	-0.3513	36.6405	3511	5	7.76	0.034	1.09
*G*. *kilimandscharicum*	O-DP-36631–36632, 36634–36635	KN1037	Kenya	Mt Kenya	-0.1461	37.3480	4019	4	7.06	0.036	2.10
*G*. *kilimandscharicum*	O-DP-36777, 36779–36792	KN1089	Kenya	Mt Kenya	-0.1416	37.3525	4135	11	10.82	0.029	1.23
*G*. *kilimandscharicum*	O-DP-36945–36949	TZ0010	Tanzania	Mt Kilimanjaro: Shira Plateau near Mt Simba	-3.0343	37.2430	3636	5	11.53	0.049	2.72
*G*. *kilimandscharicum*	O-DP-37094–37098	TZ0041	Tanzania	Mt Kilimanjaro: Shira Plateau	-3.0056	37.2416	3536	5	5.18	0.022	1.22
*G*. *kilimandscharicum*	O-DP-37770, 37771, 37774	TZ0209	Tanzania	Mt Kilimanjaro: Horombo	-3.1422	37.4407	3650	3	2.59	0.017	0.74
*G*. *kilimandscharicum*	O-DP-38183–38187	TZ0305	Tanzania	Mt Kilimanjaro: Horombo	-3.1350	37.4337	3817	5	7.29	0.032	1.46
*G*. *kilimandscharicum*	O-DP-38278–38282	TZ0327	Tanzania	Mt Kilimanjaro: Horombo	-3.1380	37.4372	3694	5	3.53	0.015	0.83
*G*. *kilimandscharicum*	O-DP-39345, 39347–39349	TZ0809	Tanzania	Mt Kilimanjaro: Masheu Point	-3.1533	37.4855	3242	4	7.76	0.039	1.91
*G*. *kilimandscharicum*	O-DP-38516–38520	TZ0384	Tanzania	Mt Meru: Saddle Hut area	-3.2170	36.7690	3594	5	8.94	0.038	1.34
*G*. *kilimandscharicum*	O-DP-38916–38917	TZ0461	Tanzania	Mt Meru: Saddle Hut area	-3.2170	36.7523	3594	5	8.71	0.039	1.70
*G*. *kilimandscharicum*	O-DP-39109–39112	TZ0501	Tanzania	Mt Meru: Betw. Saddle Hut and Miriakamba Hut	-3.2178	36.7707	3589	4	10.82	0.056	2.94
*G*. *kilimandscharicum*	O-DP-39119–39123	TZ0503	Tanzania	Mt Meru: Betw. Saddle Hut and Miriakamba Hut	-3.2178	36.7707	3589	5	12.71	0.058	2.86
***G*. *arabicum* genetic group**											
*G*. *arabicum* subsp. *arabicum*	O-DP-29336, 29339–29340	ET0036	Ethiopia	Simen Mts: Close to Gich Camp Site	13.2666	38.1078	3574	3	8.71	0.058	3.28
*G*. *arabicum* subsp. *arabicum*	O-DP-29667–29670	ET0109	Ethiopia	Simen Mts: Saha	13.2827	38.1108	3711	5	11.53	0.050	2.40
*G*. *arabicum* subsp. *arabicum*	O-DP-29778–29782	ET0139	Ethiopia	Simen Mts: Saha	13.2853	38.1184	3718	5	13.88	0.062	2.42
*G*. *arabicum* subsp. *arabicum*	O-DP-30209–30213	ET0250	Ethiopia	Simen Mts: Gich Camp Site	13.2697	38.1059	3652	5	8.24	0.037	1.94
*Geranium* sp.	O-DP-32856–32858	**ET0954**	Ethiopia	Bale Mts: Batu	6.8500	39.8532	4116	3	6.12	0.041	2.26
*Geranium* sp.	O-DP-32947–32950	**ET0974**	Ethiopia	Bale Mts: Angaso	6.9049	39.9046	3875	4	16.94	0.089	5.03
*Geranium* sp.	O-DP-33139, 33141–33143	**ET1030**	Ethiopia	Bale Mts: Angaso	6.8931	39.8974	3875	4	8.94	0.047	1.60
*G*. *arabicum* subsp.‘*Ash 1711’*	O-DP-32329–32333	ET0827	Ethiopia	Bale Mts: Sanetti, Konten	6.8448	39.8805	4129	5	8.94	0.040	1.61
*G*. *arabicum* subsp. ‘*Ash 1711’*	O-DP-34011–34015	ET1420	Ethiopia	Bale Mts: Habera	7.0187	39.7207	3484	5	12.71	0.060	2.43
*G*. *arabicum* subsp. ‘*Ash 1711’*	O-DP-34129–34133	ET1447	Ethiopia	Bale Mts: Habera	7.0073	39.7098	3482	5	11.29	0.050	2.72
*G*. *arabicum* subsp. ‘*Ash 1711’*	O-DP-34251–34255	ET1476	Ethiopia	Bale Mts: Megit	6.9907	39.6900	3499	5	8.00	0.035	2.09
*G*. *arabicum* subsp. ‘*Ash 1711’*	O-DP-34300–34301, 34303–34305	ET1485	Ethiopia	Bale Mts: Sodota	6.9897	39.7030	3520	4	4.94	0.026	1.25
*G*. *arabicum* subsp. ‘*Ash 1711’*	O-DP-33821–33825	ET1373	Ethiopia	Mt Choke	10.6382	37.8392	3908	5	8.71	0.042	2.20
*G*. *arabicum* subsp. ‘*Ash 1711’*	O-DP-43595–43599	ET1403	Ethiopia	Mt Choke	10.6575	37.8220	3919	5	14.82	0.064	2.99
*G*. *arabicum* subsp. *arabicum*	O-DP-38223–38225	TZ0316	Tanzania	Mt Kilimanjaro: Betw. Barranco and the gate	_	_	<3000	3	7.06	0.047	2.73
*G*. *arabicum* subsp. *arabicum*	O-DP-40346–40350	UG2215	Uganda	Virunga Mts: Mt Muhavura, betw. 2nd Hut and summit	-1.3813	29.6760	4050	5	12.94	0.061	2.78
*G*. *arabicum* subsp. *arabicum*	O-DP-40524–40528	UG2251	Uganda	Ruwenzori Mts: Lower Bigo Valley	0.3850	29.9273	3425	5	19.29	0.090	5.49
*G*. *arabicum* subsp. *arabicum*	O-DP-41060–41064	UG2388	Uganda	Ruwenzori Mts: Bukurungu Valley	_	_	3925	5	5.88	0.028	1.31

### AFLP analysis

DNA was extracted from silica-dried leaf tissue using a GeneMole® robot and the Mole StripTM Plant DNA Kit (QIAGEN, Nordic, Oslo, Norway) or DNeasyTM Plant Mini Kit (QIAGEN, Valencia, CA). Leaf tissue was ground in 2.0 μL tubes with two tungsten carbide beads for 2 min at 15 Hz in a mixer mill (MM301, Retsch GmbH & Co., Haan, Germany), after which 250 μL of lysis buffer was added, vortexed, spun briefly, incubated on a heat block for 10 min at 65°C, and centrifuged at 14000 Hz for 2 min. 200 μL of the lysate was transferred to new tubes and loaded to the robot, which was set to produce a final elution volume of 100 μL.

About 10% of the samples were extracted twice to test for reproducibility of the markersfollowing [12]. AFLP data was generated following [13],except that the PCR reaction volumes were reduced by 50% and pre-selective PCR products were diluted ten times. Twelve primers pairs were initially tested on one plant from each of eight mountains, and the following three primer combinations, which resulted in many polymorphic and well separated bands, were selected for the final analysis:6FAM-*Eco*RI-ATG/*Mse*I-CGA, VIC-*Eco*RI-ACA/*Mse*I-CAC, and NED-*Eco*RI-AGC/*Mse*I-CTG. For each sample, 2.0 μL 6-FAM, 2.0μL VIC and 3.0μL NED labeled selective PCR products were mixed and added to a master mix of 11.7 μL formamide and 0.3μL GENESCAN ROX 500 internal-lane size standard, denatured at 95°C for 5 min and cooled on ice before run on an ABI3100 sequencer (Applied Biosystems, Foster City, USA).

### Data analyses

Markers in the size range 50–500 base pairs (bp)were scored as present (1) or absent (0) using GeneMapper^®^ version 4.0 (Applied Biosystems, Foster City, USA).Peaks of low intensity were only scored when unambiguous. Error rate calculation and data cleaning [12] were done separately for each primer combination, and duplicates were removed and matrices combined prior to further analyses. Nei’s gene diversity (*D*; estimated as the average proportion of pairwise differences among genotypes; [10]), proportion of polymorphic markers (*P*%), and genetic distinctiveness or rarity (DW; estimated as frequency-down-weighted markers; [11]) were calculated using AFLPdat [14]. Pairwise genetic similarity between AFLP phenotypes was estimated using Dice’s coefficient of similarity in NTSYSpc 2.1 [15] and visualized using Principal Coordinate Analyses (PCoAs).

Bayesian clustering was carried out in STRUCTURE v 2.3.3 [16]. We used the recessive allele model to accommodate the dominant nature of the AFLP markers [17] and compared the no admixture model with uncorrelated allele frequencies vs the admixture model with correlated allele frequencies. Based on the result from the preliminary analysis, we selected the admixture model with correlated allele frequency for the final analysis. Analyses were performed at the Lifeportal, University of Oslo (http://www.lifeportal.uio.no). Analyses were run with *K* ranging from 1–10, and for each *K*, 10 replicate runs with a burn-in period of 200,000 and 1,000,000 iterations were used. We used the R-script STRUCTURE-SUM to summarize the results. Log probability of the data, *L*(*K*), as a function of *K* ranging from 1to 10 and the rate of change in the probability between successive runs, *ΔK*, were calculated according to [18]. In addition, similarity among different runs for the same *K* was estimated according to[19]. We used the program CLUMPP [20] to estimate the average individual admixture value among the replicated runs for the selected optimal *K*, and DISTRUCT [21] to graphically visualize the clustering.

A Neighbor-Net diagram [22] was constructed based on uncorrected *p*-distance using SplitsTree4 v. 4.12.6 [23], and support for branches was estimated from 1000 bootstrap replicates using TreeCon version 1.3b [24]. Analyses of Molecular Variance (AMOVAs) were performed to investigate partitioning of genetic variation at hierarchical and non-hierarchical levels using ARLEQUIN v. 3 [25]. Significance of genetic differentiation and pairwise population differentiation among mountains were estimated with 1000 permutations. We tested the correlation between geographic and genetic (*F*ST) pairwise distances for the total dataset and for each of the two genetic groups separately, using Mantel test implemented in GeneAlex 6.5[26], using 1000 iterations.

## Results

**Fig 2:**
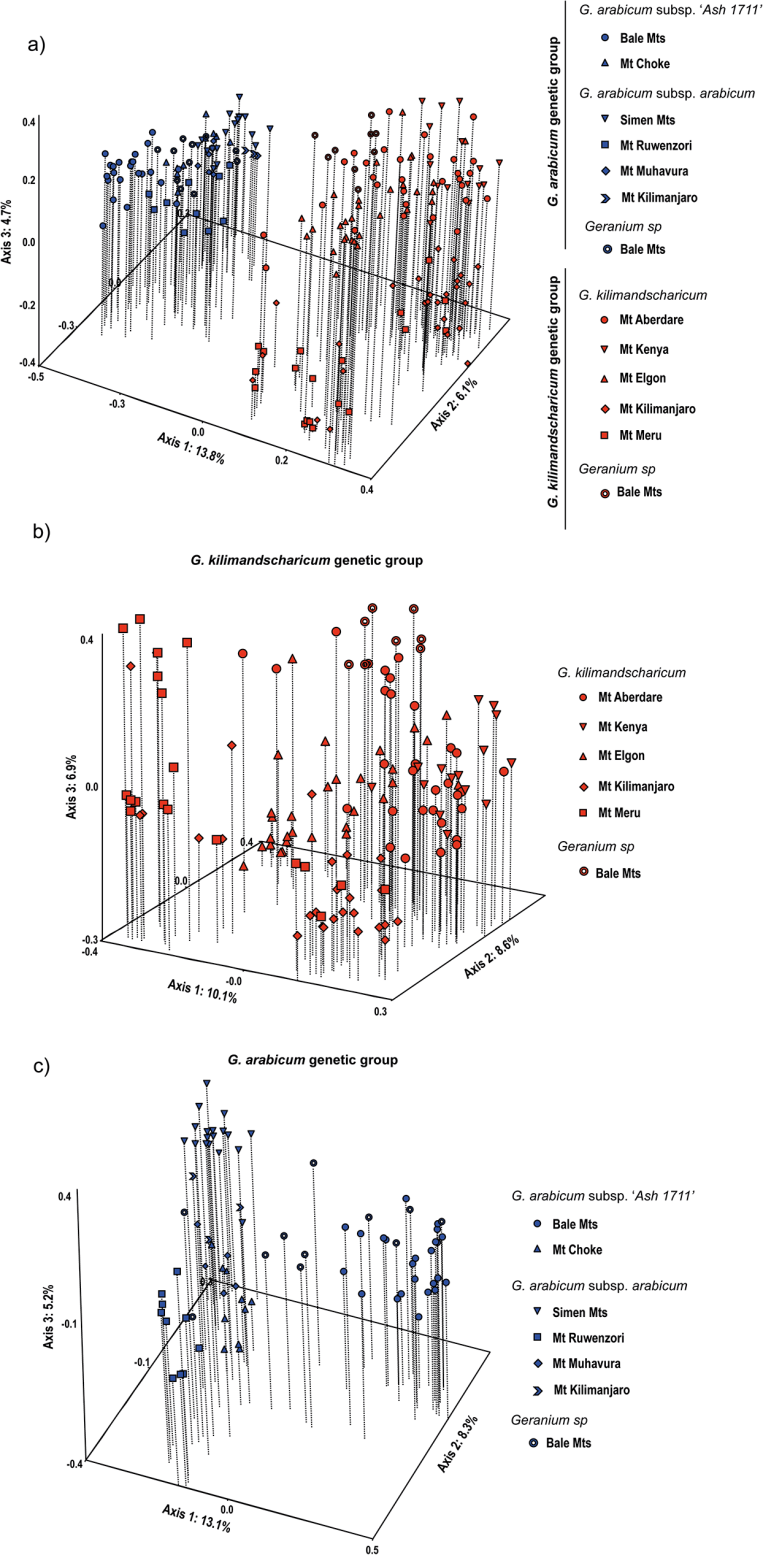
Principal Coordinates Analyses (PCoA) based on Dice’s coefficient of similarity among AFLP phenotypes observedin eastern African high-altitude populations of *Geranium*. a) Total datasetincluding all 45 populations, with colours representing the two main genetic groups inferred in the STRUCTURE analyses (blue: *G*. *arabicum* group, red: *G*. *kilimandscharicum* group). b) Subset of the 30 populations belonging to the *G*. *kilimandscharium*group. c) Subset of the 15 populations belonging to the *G*. *arabicum*group.

**Fig 3:**
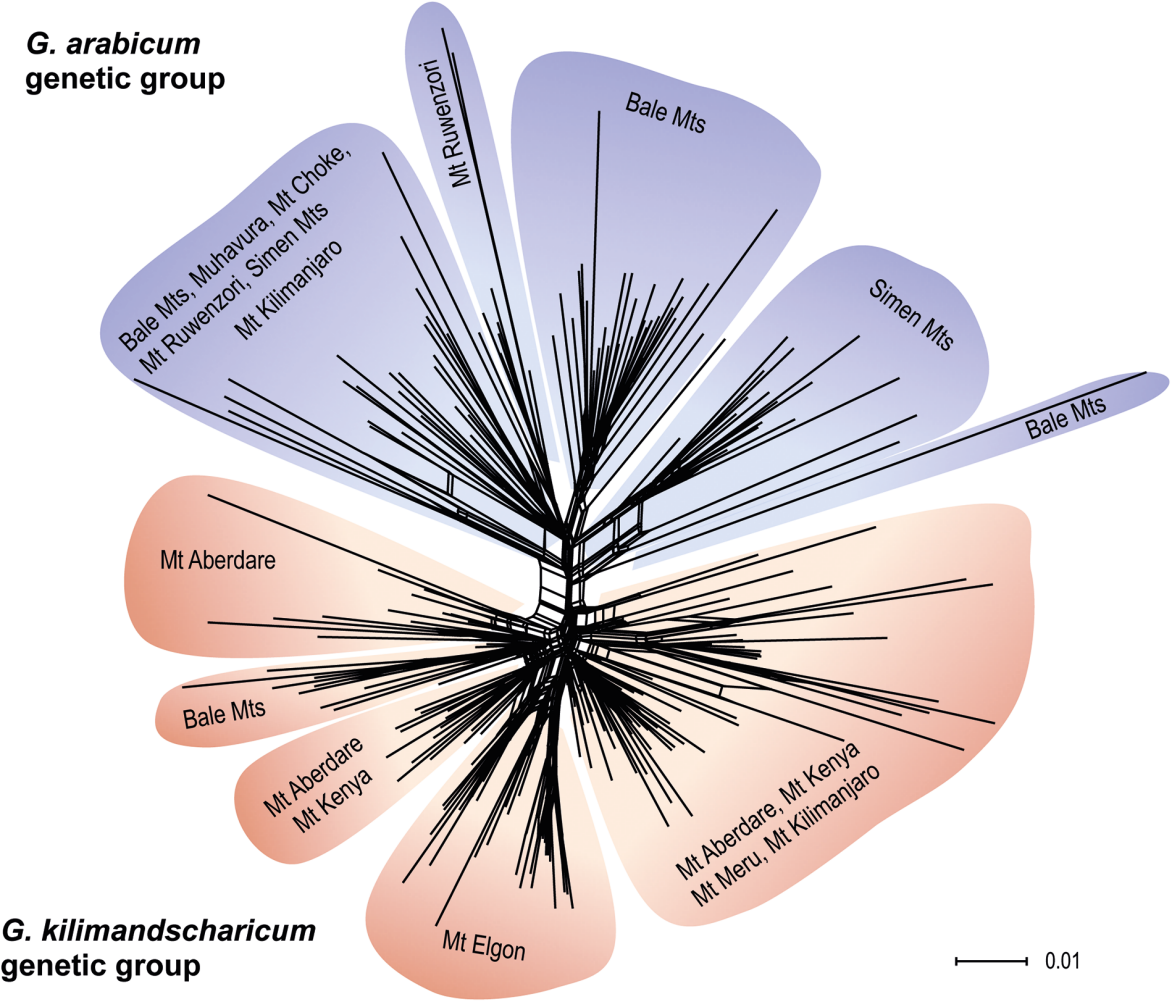
Neighbor-Net diagram based on uncorrected p-distances among the AFLP phenotypes observed in eastern African high-altitude populations of *Geranium*. Colours represent the two main genetic groups inferred in the STRUCTURE analyses (blue: *G*. *arabicum* group, red: *G*. *kilimandscharicum* group). No major branch obtained bootstrap support greater than 50%.

A total of 211 samples and 425 markers, of which 306 (72%) were polymorphic, were kept after cleaning the data. Reproducibility of the markers was 98.1%. In the STRUCTURE analyses, the change in the log probability of the data, L(*K*), showed highest increment from *K* = 1 to *K* = 2 (S1A Fig). The rate of change in the probability between successive *K*s, Delta*K*, indicated a single, highest peak at *K* = 2 (S1B Fig), and the similarity among the replicated runs also revealed highest convergence at this point (S1C Fig). We therefore inferredthe optimal partitioning of the data to be intotwo genetic groupsfrom the STRUCTURE analyses (*K* = 2; Figs 1–3).The *G*. *kilimandscharicum* group included all populations referred to the eastern East African endemic *G*. *kilimandscharicum* (all populations from Mt. Kenya, Mt. Aberdare, Mt. Elgon and Mt. Meru, and all but one from Mt. Kilimanjaro), as well as some of the plants from the Ethiopian Bale Mts referred to *Geranium* sp. The *G*. *arabicum* group comprised all populations referred to the widespread *G*. *arabicum* (all populations from the western East African mountains Muhavura and Ruwenzori and the Ethiopian Simen and Choke mountains, most populations from the Ethiopian Bale Mts, and the single montane forest populationfrom Mt Kilimanjaro), as well as the remaining Bale Mts plants referred to *Geranium* sp. The plants from the four populations (ET0677, ET0954, ET0974 and ET1030) referred to *Geranium* sp. were accordingly divided between the two genetic groups, with one complete population (ET0677) and four individual plants from the three other populations placed in the *G*. *arabicum*group, and with the remaining plants placed in the *G*. *kilimandscharicum* group (Table 1). All of the individual *Geranium* sp. plants showed however some degree of admixture (Fig 1). Some degree of admixture was also found between the two genetic groups in other mountains.

Similar genetic structuring was observed in the PCoA and Neighbor-Net analyses. The two genetic groups inferred from the STRUCTURE analyses were distinguished along the first axis of the PCoA of the total dataset, explaining 13.8% of the total variation (Fig 2A). Also in this analysis, the Bale Mts plants referred to *Geranium* sp. were divided between the two groups. Separate PCoAs of each genetic group showed large and continuous variation within each of them. In the *G*. *kilimandscharicum* group, there was no clear differentiation among mountains, and the *Geranium* sp. plants grouped closely with plants from Mt Aberdare (Fig 2B). In the *G*. *arabicum* group, the two subspecies could not beconsistently separated. The Bale plants referred to ssp. ‘*Ash 1711*’ were placed at one extreme of axis 1, whereas the Choke plants referred to the same subspecies grouped close to ssp. *arabicum*plants from the Simen Mts (Fig 2C). The mountains were however more clearly differentiated in this genetic group. Axis 1 corresponded to a division across the Rift Valley in Ethiopia (Bale Mts vs Simen/Choke Mts), and most of the East African plants were separated along axis 2. The Neighbor-Net analysis (Fig 3) was more or less star-shaped and without strongly supported major branches, but with the most distinct division corresponding to the two genetic groups inferred from the STRUCTURE analyses.

**Fig 4:**
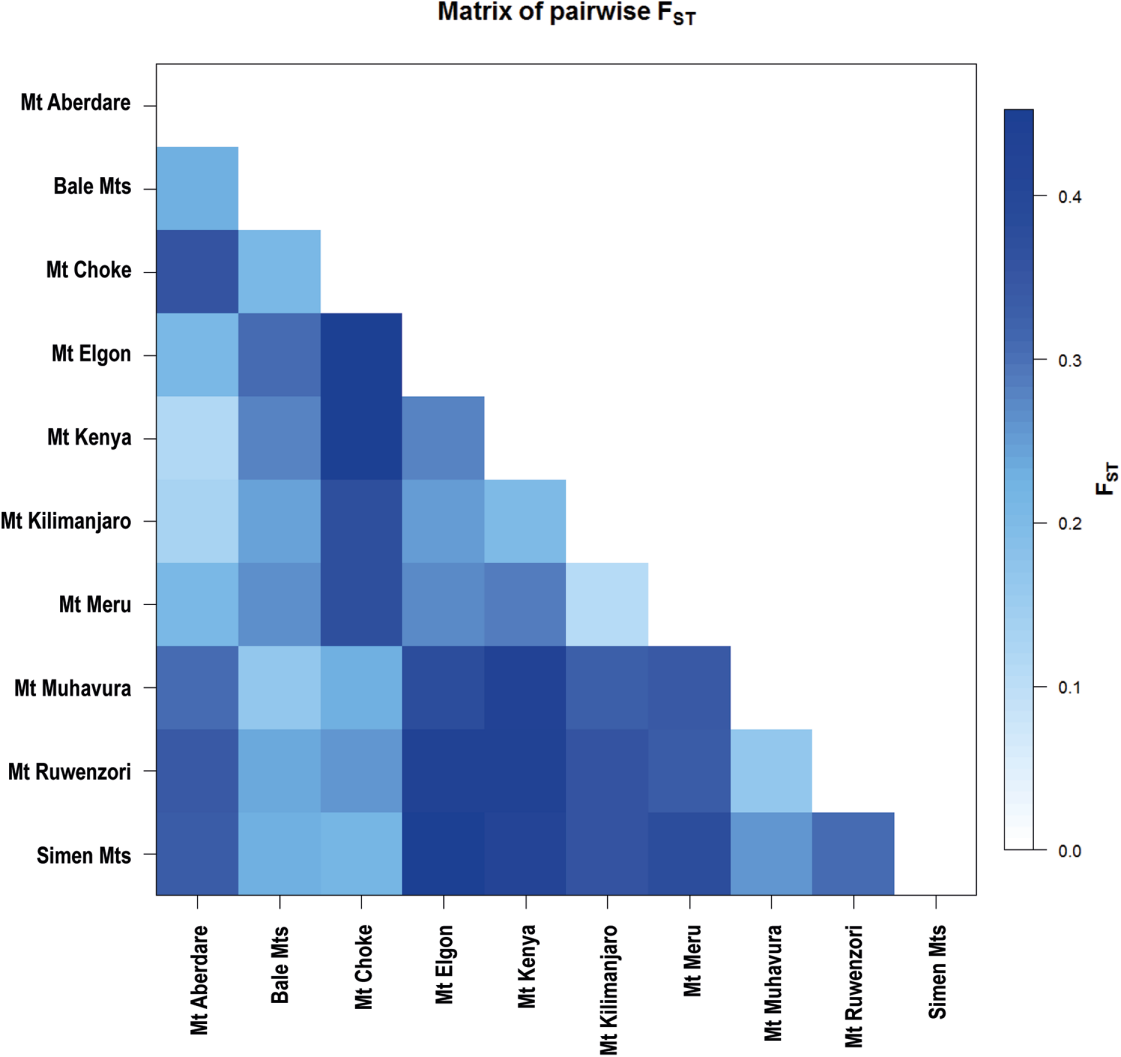
Pairwise population differentiation (estimated as *F*_ST_, equiv. to *Φ*_ST_) among the eastern African high-altitude populations of *Geranium* on ten mountains based on AFLP data for 211individual plants from 45 populations.

The *G*. *arabicum* genetic group had higher average within-population gene diversity (*D* = 0.072) and higher rarity (DW = 2.68) than the *G*. *kilimandscharicum*genetic group (*D* = 0.051; DW = 1.75; Table 2). The highest diversity and rarity were recorded in the Mt Ruwenzori and Mt Muhavura populations of *G*. *Arabicum*(*D* = 0.061–0.066; DW = 3.20–3.62). Very little diversity was recorded in the Mt Kilimanjaro and Mt Kenya populations of *G*. *Kilimandscharicum*(*D* = 0.031–0.035; Table 2). In the non-hierarchical AMOVA analysis, most of the genetic variation was found within populations (62.32%; Table 3). In hierarchical AMOVAs, 21.53% of the variation was found between the two genetic groups, 24.42% among populations within groups, and 54.05% within populations. When dividing the data according to the three species inferred from morphology, 20.05% of the variation was found among the three groups, 24.65% among populations, and 55.30% within populations.Within each genetic group, much higher proportions of the genetic variation werefound within (66.87% and 71.14%) than among populations (33.13% and 28.86%, *F*_ST_ = 0.3313 and 0.2886). We found highly significant (*P*< 0.001) genetic differentiation among the ten mountains, with the strongest divergence between the Simen Mts and Mt Elgon (Fig 4). We also found a significant pattern of isolation by distance in the Mantel test for the total dataset (r = 0.4113, S2A Fig) and within each of the two genetic groups (r = 0.5710and r = 0.4719, for *G*. *kilimandscharicum* S2B Fig and *G*. *arabicum* S2C Fig, respectively, *P*< 0.001 for all values).

**Table 2:** Gene diversity and genetic rarity based on AFLP data for eastern African high-altitude populations of *Geranium* pulled by genetic group and by mountain.*P*(*%*): Percentage of polymorphic loci; *D*: Nei’s gene diversity following [10]; DW: frequency-down-weighted marker value as a measure of genetic rarity following [11].

Geneticgroup	Mountain	*n*	*P* (*%*)	*D*	DW
*G*. *kilimandscharicum*		126	74.82	0.051	1.75
	Bale Mts	5	8.94	0.041	2.99
	Mt Elgon	30	22.35	0.041	1.24
	Mt Aberdare	30	35.29	0.044	1.96
	Mt Kenya	15	14.59	0.031	1.56
	Mt Kilimanjaro	27	24.94	0.035	1.59
	Mt Meru	19	26.82	0.052	2.24
*G*. *arabicum*		70	68.24	0.072	2.68
	Simen Mts	18	26.82	0.054	2.59
	Mt Choke	10	18.12	0.055	2.68
	Bale Mts	24	28.24	0.047	2.21
	Mt Kilimanjaro	3	7.06	0.047	2.94
	Mt Muhavura	5	12.94	0.061	3.20
	Mt Ruwenzori	10	23.76	0.066	3.62

**Table 3:** Analyses of molecular variance (AMOVAs) based on AFLP data for 45 eastern African high-altitude populations of *Geranium*, including26 populations tentatively referred to *G*. *kilimandscharicum*,four populations to *Geranium*sp. sensu [8], and 15 populations to *G*. *arabicum* based on morphology. *F*-statistics are provided for genetic differentiation among groups (*F*_CT_), among populations within groups (*F*_SC_), and among all populations (*F*_ST_). All values were significant at *P*<0.0001.

Source of variation	*df*	Variance components	Percentage of variation	*F*-statistics
Among populations	44	5.45	37.68	*F*_ST_ = 0.3768
Within populations	166	9.02	62.32	
Among three morphological groups	2	3.19	20.05	*F*_CT_ = 0.1923
Among populations	43	3.92	24.65	*F*_SC_ = 0.3129
Within populations	163	8.79	55.30	*F*_ST_ = 0.4450
Between two genetic groups*	1	3.50	21.53	*F*_CT_ = 0.2153
Among populations	44	3.97	24.42	*F*_SC_ = 0.3112
Within populations	163	8.80	54.05	*F*_ST_ = 0.4595
Within *G*. *kilimandscharicum* genetic group*				
Among populations	27	3.67	33.13	*F*_ST_ = 0.3313
Within populations	100	7.41	66.87	
Within *G*. *arabicum* genetic group				
Among populations	17	4.46	28.86	*F*_ST_ = 0.2886
Within populations	63	10.99	71.14	

*Two individuals each representing a single population were removed from the analysis.

## Discussion

Using genome-wide genetic markers and range-wide sampling, we have shown that the high-alpine populations of the *Geranium arabicum/kilimandscharicum* complex in the eastern African mountains present quite simple genetic structuring, in spite of their intricate morphological variation which recently led to the description of two new endemic taxa. We identified only two genetic groups, of which one is widespread (*G*. *arabicum*), and the other seem to have evolved as a specialized high-alpine ecotype in one group of mountains but not elsewhere (*G*. *kilimandscharicum* in eastern East Africa). It appears that the morphological complexity and taxonomic problems in the group have been caused by a combination of four processes: by differentiation into one low-alpine and one high-alpine ecotype in eastern East Africa, by local hybridization between these ecotypes, by at least one episode of long-distance dispersal of the high-alpine ecotype followed by hybridization and establishment of admixed plants in naturally disturbed habitats, and by phenotypic plasticity or parallel evolution in some leaf characters. In the following, we discuss the available evidence for the influence of each of these processes.

Morphological differentiation among the low-altitude and high-altitude populations growing in the same mountains in eastern East Africa has long been recognized e.g. [9]; [7], and the observation of occasional morphological intermediates led [7] to suggest that introgression between them occurs at intermediate altitudes. His hypothesis is supported by our data. Although our sampling was concentrated at high altitudes as part of a larger project restricted to the alpine zone, we sampled one montane forest population on Mt Kilimanjaro that clearly belonged to *G*. *arabicum* based on both morphology and AFLP data (Figs 1–3). It nevertheless showed some admixture with *G*. *kilimandscharicum*. Several of the *G*. *kilimandscharicum* plants sampled from each of the other eastern East African mountains also showed some degree of admixture with *G*. *arabicum* (Fig 1). The fact that only modest morphological and genetic divergence occurs between the two taxonomically recognized species, and due to their sympatric occurrence on the eastern East African mountains as well as the apparently frequent introgressive hybridization, we tend to agree with the suggestion of [8] to recognize the high-alpine *G*. *kilimandscharicum* as a subspecies of the more widespread and morphologically variable *G*. *arabicum*.

The conspicuous and puzzling morphology of the high-alpine populations referred to the tentative new species endemic to the Bale Mountains in Ethiopia seems to be caused by hybridization between *G*. *arabicum* and *G*. *kilimandscharicum*. These populations are restricted to habitats that are naturally disturbed, typically by the giant mole rat. While other mole rats feed underground, the giant mole rat that is endemic to the Bale Mountains mostly forages above ground, heavily disturbing the soil and clearing the vegetation around their tunnel openings [27]. This creates a type of habitat well-known to be suitable for establishment of plant hybrids, e.g. [28]. We found that all individual plants referred to *Geranium* sp. showed some degree of admixture between the local *G*. *arabicum* and the East African *G*. *kilimandscharicum*, suggesting that they originated by hybridization after long-distance dispersal of *G*. *kilimandscharicum* from East Africa to the Bale Mountains. Because *G*. *kilimandscharicum* is exclusively high-alpine in East Africa, we consider gradual migration of this ecotype across the vast lower-lying Kenyan gap to Ethiopiato be unlikely even under colder climatic periods, when the alpine belt may have been shifted downwards by 1000 m [29]. Notably, long-distance dispersal across the Kenyan gap has also been inferred based on genetic data for several other afro-alpine species ([4–5];). The plants referred to *Geranium* sp. in our sampling do not seem to represent F_1_ hybrids, but rather segregating later-generation hybrids or backcrosses, as inferred from their variable assignments to the two genetic groups (Fig 1). Long-distance dispersal of the high-alpine *G*. *kilimandscharicum*seems also to have occurred to other mountains, as inferred from the occurrence of some admixed plants in the western Rift mountains (Ruwenzori and Muhavura) and the Ethiopian Simen Mts (Fig 1).

The high-alpine Ethiopian populations referred to the tentative new subspecies of *G*. *arabicum*, ssp. ‘*Ash 1711*’, grouped with ssp. *arabicum* according to their geographic origin rather than morphology (Fig 2C). This pattern suggests that the leaf characters used to separate this subspecies from ssp. *arabicum*, leaf outline and shape of leaf lobes, are to some degree phenotypically plastic or subjected to parallel evolution, adding further taxonomic confusion to this plant complex.

The lack of geographic structuring we observed in the high-alpine *G*. *kilimandscharicum* across the widely separated eastern Rift mountains (Fig 2B) suggests a recent history of colonization of the different mountains or extensive intermountain gene flow. This finding, as well as the evidence for admixture with *G*. *arabicum* in other distant mountain groups, suggest that *G*. *kilimandscharicum* has a considerable capacity for long-distance dispersal even if it is obviously adapted to short-distance dispersal in terms of morphology. High capacity for long-distance dispersal in plants adapted to short-distance dispersal has also been inferred in some other afro-alpine plants (Wondimu *et al*., 2014) and in many arctic-alpine plant species [30–31]. In contrast, genetic diversity was more distinctly structured according to mountains in *G*. *arabicum* (Fig 2C), which has similar dispersal adaptations. The Rift Valley in Ethiopia appears as a barrier in *G*. *arabicum*, as also found for other species [32]; [29]; [33]; [14]. This result is surprising because *G*. *arabicum* is able to grow at lower altitudes and therefore could have been thought to cross lowland barriers via gradual migration during colder climates. It appears that the mode of dispersal and lower limit of the altitudinal range cannot always be used as predictors of the strength of intermountain barriers against gene flow in the afro-alpine flora.

## Supporting information

S1 Fig Summary results of STRUCTURE analyses. a) Log likelihood of the data,L(*K*), as a function of *K* ranging from 1 to 10. b) Mean Delta*K* for the rate of change in the probability between successive runs, Delta*K* as a function of *K*, calculated according to [33]. c) Average similarity coefficients for the pairwise comparisons among 10 runs for a given K.
(DOCX)

S2 Fig Mantel test for correlation between pairwise genetic-distance (Fst) and geographic-distance (*P*< 0.001; 1000 permutation). a) for the total dataset, b) *G*. *kilimandscharicum* and c) *G*. *arabicum* genetic groups.
(DOCX)
