# Supplementary material for: Genetic structuring, dispersal and taxonomy of the high-alpine populations of the Geranium arabicum/kilimandscharicum complex in tropical eastern Africa
Source: PLoS One. 2017 May 26;12(5):e0178208. doi: 10.1371/journal.pone.0178208 (PMC5446165; doi:10.1371/journal.pone.0178208)
Supplement: S1 Fig — a) Log likelihood of the data,L(K), as a function of K ranging from 1 to 10. b) Mean DeltaK for the rate of change in the probability between successive runs, DeltaK as a function of K, calculated according to [33]. c) Average similarity coefficients for the pairwise comparisons among 10 runs for a given K. (DOCX) [file pone.0178208.s001.docx]

Supporting Information 1

**S1a Fig**

**S1b Fig**

**S1c Fig**

**References**

Evanno G, Regnaut S, Goudet J. Detecting the number of clusters of individuals using the software STRUCTURE: simulation study. Mol. Ecol. 2005; 14:2611–2620

Rosenberg NA, Pritchard JK, Weber JL, Cann HM, Kidd KK, Zhivotovsky LA, Feldman MV.Genetic structure of human populations. Science 2002; 298:2381–2385
