# Supplementary figures and images for: Genetic structuring, dispersal and taxonomy of the high-alpine populations of the Geranium arabicum/kilimandscharicum complex in tropical eastern Africa
Source: PLoS One. 2017 May 26;12(5):e0178208. doi: 10.1371/journal.pone.0178208 (PMC5446165; doi:10.1371/journal.pone.0178208)

Supporting Information 2

**S2a Fig**


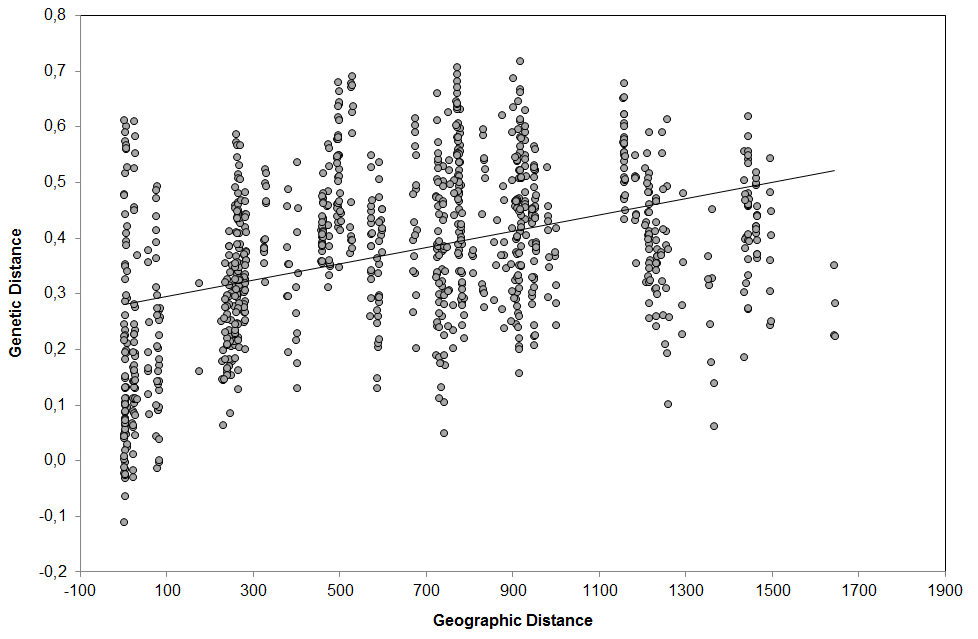


**S2b Fig**


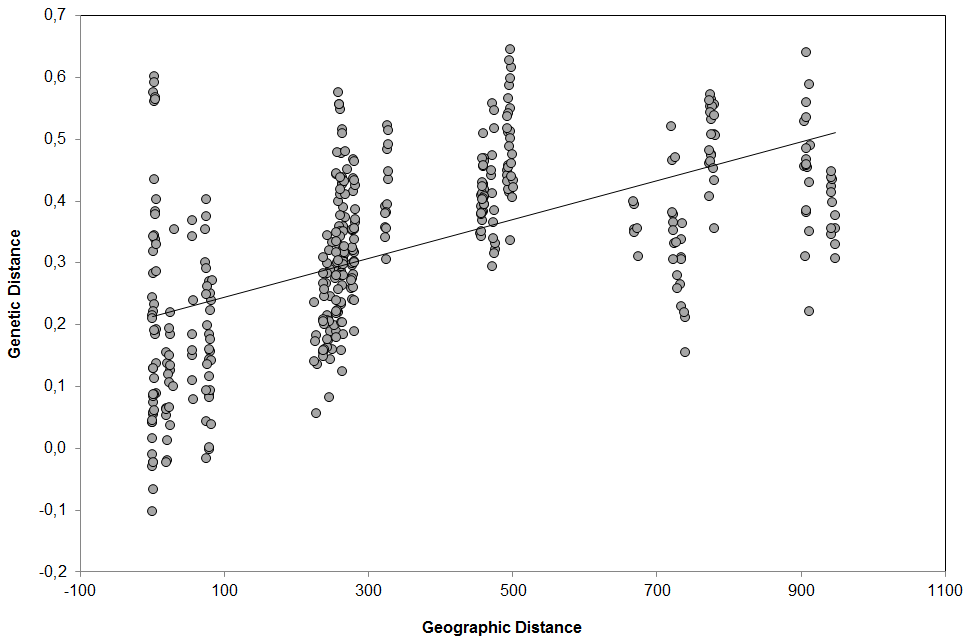


**S2 c Fig**


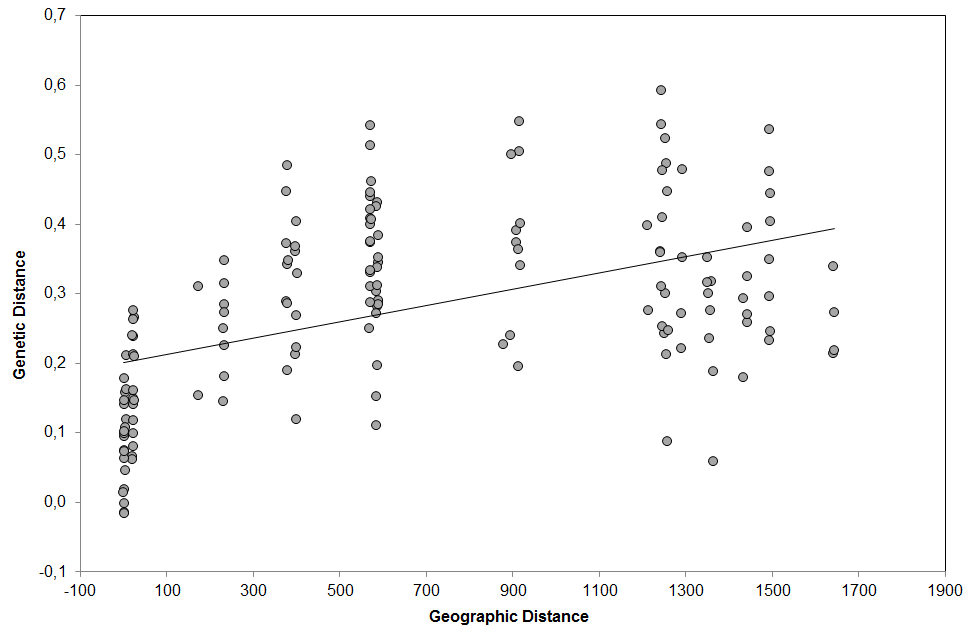

Supplement: S2 Fig — a) for the total dataset, b) G. kilimandscharicum and c) G. arabicum genetic groups. (DOCX) [file pone.0178208.s002.docx]
